# Assessment of Prediction Confidence and Domain Extrapolation of Two Structure–Activity Relationship Models for Predicting Estrogen Receptor Binding Activity

**DOI:** 10.1289/txg.7125

**Published:** 2004-07-16

**Authors:** Weida Tong, Qian Xie, Huixiao Hong, Leming Shi, Hong Fang, Roger Perkins

**Affiliations:** ^1^Center for Toxicoinformatics, and; ^2^Bioinformatics Laboratory, National Center for Toxicological Research, Food and Drug Administration, Jefferson, Arkansas, USA

**Keywords:** applicability domain, Decision Forest, domain extrapolation, EDCs, endocrine-disrupting chemicals, estrogen receptor binding, QSAR, prediction confidence, regulatory application

## Abstract

Quantitative structure–activity relationship (QSAR) methods have been widely applied in drug discovery, lead optimization, toxicity prediction, and regulatory decisions. Despite major advances in algorithms and software, QSAR models have inherent limitations associated with a size and chemical-structure diversity of the training set, experimental error, and many characteristics of structure representation and correlation algorithms. Whereas excellent fit to the training data may be readily attainable, often models fail to predict accurately chemicals that are outside their domain of applicability. A QSAR’s utility and, in the case of regulatory decisions, justification for usage increasingly depend on the ability to quantify a model’s potential for predicting unknown chemicals with some known degree of certainty. It is never possible to predict an unknown chemical with absolute certainty. Here we report on two QSAR models based on different data sets for classification of chemicals according to their ability to bind to the estrogen receptor. The models were developed by using a novel QSAR method, Decision Forest, which combines the results of multiple heterogeneous but comparable Decision Tree models to produce a consensus prediction. We used an extensive cross-validation process to define an applicability domain for model predictions based on two quantitative measures: prediction confidence and domain extrapolation*.* Together, these measures quantify the accuracy of each prediction within and outside of the training domain. Despite being based on large and diverse training sets, both QSAR models had poor accuracy for chemicals within the domain of low confidence, whereas good accuracy was obtained for those within the domain of high confidence. For prediction in the high confidence domain, accuracy was inversely proportional to the degree of domain extrapolation. The model with a larger training set of 1,092, compared with 232 for the other, was more accurate in predicting chemicals at larger domain extrapolation, and could be particularly useful for rapidly prioritizing potential endocrine disruptors from large chemical universe.

Quantitative structure–activity relationships (QSARs) have been extensively applied in a broad range of scientific areas, including chemistry, biology and toxicology (Hansch et al. 1995a ,1995b). QSAR is now an inexorably imbedded tool in drug development, from lead discovery to lead optimization (Hopfinger and Tokarski 1997; Kubinyi et al. 1998). There is increasing use of QSAR early in the drug discovery process as a screening and enrichment tool to eliminate from further development those chemicals lacking drug-like properties (Lipinski et al. 1997) or those chemicals predicted to elicit a toxic response. The availability of powerful new algorithms and scientists trained in their usage suggests the eventual common use of QSAR beyond the pharmaceutical industry to human and environmental regulatory authorities (Benigni and Richard 1998; Bradbury 1994; Hansch et al. 1995a, 1995b; Russom et al. 1995; Schultz and Seward 2000; Tong et al. 2002 ,2003a).

Any QSAR model produces some degree of error. This is partially due to the inherent limitation in predicting a biological activity based solely on the chemical structure. One can argue from the principles of chemistry that molecular structure of a chemical is the key to understanding its physicochemical properties and ultimately its biological activity and the influence on organisms (Johnson and Maggiora 1990). However, biological activity of a chemical is an induced response that is influenced by numerous factors dictated by the levels of biological complexity of the system under investigation. The relationship between structure and activity is thus more implicit and thereby requires a more thorough investigation and rigorous validation (Tong et al., 2004).

Application of QSARs in regulation has proven to be cost effective for prioritizing untested chemicals for more extensive and costly experimental evaluation. However, for QSARs to be accepted by the regulatory communities, their limitation for use needs to be identified. This is important because a QSAR model’s ability to predict unknown chemicals depends largely on the nature of the training set and the algorithm used to establish the structure–activity relationship (Eriksson et al. 2003). A model’s predictive accuracy and confidence for different unknown chemicals varies according to how well the training set represents the unknown chemicals and how robust the model is in extrapolating beyond the chemistry space defined by the training set (i.e., training domain). Therefore, assessing a model’s “prediction confidence,” defined as the certainty for a prediction, and “domain extrapolation,” defined as the prediction accuracy outside the training domain, is a vital step toward defining the application domain of a model for the regulatory acceptance of QSARs.

A large number of environmental chemicals known as endocrine-disrupting chemicals (EDCs) are suspected of disrupting endocrine functions by mimicking or antagonizing natural hormones in experimental animals, wildlife, and humans (Hileman 1997). EDCs may exert adverse effects through a variety of mechanisms, including estrogen receptor (ER)–mediated mechanisms of toxicity (Fang et al. 2003b). Accordingly, the U.S. Congress in 1996 mandated that the U.S. Environmental Protection Agency (EPA) develop a strategy for screening and testing a large number of chemicals found in drinking water (Safe Drinking Water Act 1996), and food additives (Food Quality Protection Act 1996) for their endocrine disruption potential. Consequently, more than 58,000 environmental and industrial chemicals have been identified as candidates for possible experimental testing. QSARs could be used as an inexpensive prescreening tool to prioritize the chemicals for further testing (Tong et al. 2002).

In this article, we applied a novel consensus QSAR method, called Decision Forest (DF) (Tong et al. 2003b), to classify chemicals into active and inactive categories of ER binding as a priority-setting tool for EDCs. We assessed the applicability domain of the DF models through characterizing the prediction confidence and domain extrapolation for predicting unknown chemicals.

## Material and Methods

### Estrogen Receptor Data Sets and Structural Descriptors

Two data sets were used, and the ER binding activity for both data sets was obtained from the competitive ER binding assay (Blair et al. 2000; Branham et al. 2002). The first data set, designated ER232, contained 232 chemicals, 131 active, and 101 inactive that were tested in our lab (Fang et al. 2003a). This data set has been extensively used by others and us to develop SAR/QSAR models for predicting ER binding activity (Hong et al. 2002; Shi et al. 2001, 2002; Tong et al. 2002, 2003c). The second data set, designated ER1092, is an aggregation of data from the literature containing 1,092 chemicals, of which 350 are active and 736 are inactive. Inactive means that no activity was detectable in the assay. Both data sets span a wide range of structural diversity and activity.

Because a previous study indicated no significant difference in results between two-dimensional (2D) descriptors and 3D descriptors in DF (Tong et al. 2003b), only 2D descriptors were used in this study, and these were computed using Molconn-Z, version 4.07 (http://www.eslc.vabiotech.com/molconn/). After removing descriptors that were constant across all chemicals in a data set, more than 270 descriptors remained and were used in model development.

**Figure 1:**
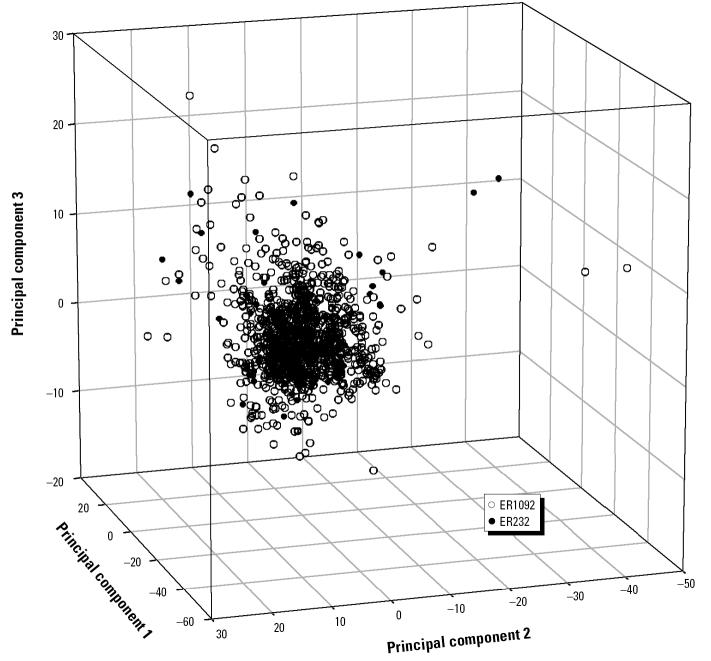
Comparison of structural diversity of ER232 and ER1092 in a chemistry space defined by three principal components of over 270 2D structural descriptors.

The structural diversity of both data sets was compared in the chemistry space defined by the 2D descriptors on the first three principle components plot (Figure 1). Not surprisingly, ER1092 was found to span much greater structural diversity than ER232.

### Decision Forest

DF is a consensus modeling technique (Tong et al. 2003b) that combines multiple Decision Tree models (hereafter called trees) in a manner that results in more accurate predictions. Because combining several identical trees produces no gain, the rationale behind DF is use of individual trees that are different (i.e., heterogeneous) yet comparable in their prediction accuracy to represent the association of structure and biological activity. The heterogeneity requirement assures that each tree uniquely contributes to the combined prediction, whereas the quality comparability requirement assures that each tree contributes equally to the combined prediction. Because a certain degree of noise is always present in biological data, optimizing a tree inherently risks overfitting the noise. DF attempts to minimize overfitting by maximizing the difference among individual trees to cancel some random noise through combining the trees. The maximum difference was achieved by constructing each individual tree using a distinct set of descriptors.

Details of the DF algorithm have been reported by Tong et al. (2003b). Briefly, developing a DF model (called forest hereafter) comprises four steps: *a*) construct and prune a tree; *b*) develop the next tree based on only the descriptors that have not been used in the previous tree(s); *c*) repeat steps 1 and 2 until no more trees can be developed; *d*) classify (i.e., predict) a chemical based on the results of all trees.

Each tree in a forest is developed using a variant of the Classification and Regression Tree (CART) method (Breiman et al. 1995) that has two steps: *a*) tree construction and *b*) tree pruning. During tree construction, the algorithm identifies the descriptors that best divide the chemicals in the parent node into two child nodes. The split maximizes the homogeneity of the activity population in each child node (e.g., one node predominately contains active chemicals, whereas the other predominately contains inactive chemicals). Then, the child nodes become parent nodes for further splits and splitting continues until chemicals in each node are either in one classification category or cannot be split further to improve the quality of the tree. To avoid overfitting the training data, the tree is then cut down to a desired size using tree cost-complexity pruning (Clark and Pregibon 1997). At the end, the terminal node of each tree generally is populated by different ratios of active versus inactive chemicals.

In each tree, the probability (0–1) for an “unknown” chemical to be active is taken to be the percentage of active chemicals in the terminal node to which the chemical belongs. The mean probability value for a chemical in all trees in the forest is calculated to assign the classification of the chemical. Chemicals that have a mean probability > 0.5 are designated active, whereas those that have a mean probability < 0.5 are designated inactive.

### Prediction Confidence

Past results have shown that DF predictions are of high confidence for active chemicals with a large probability value (approaching 1) and for inactive chemicals with low probability value (approaching zero), whereas the low confidence predictions are mostly found for chemicals with probability approaching 0.5 (Tong et al. 2003b). Based on this observation, the following equation was used to calculate the confidence level of a prediction:


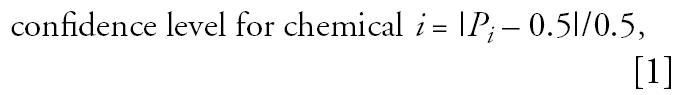


where *P_i_* is the probability value for chemical *i*. In this equation, the confidence associated with active and inactive prediction is scaled in parallel to the range between zero and 1. If we assume that a high confidence prediction is defined as confidence level > 0.4, both probability ranges of 0.0–0.3 and 0.7–1.0 will be considered the high confidence (HC) region, and 0.3–0.7 is the low confidence (LC) region. In other words, a high prediction certainty is expected when a chemical with predicted probability in the range 0.0–0.3 is classified as inactive, or when a chemical with probability in the range 0.7–1.0 is predicted as active. In contrast, prediction confidence is lower for chemicals with probabilities in the range 0.3–0.7.

### Domain Extrapolation

**Figure 2:**
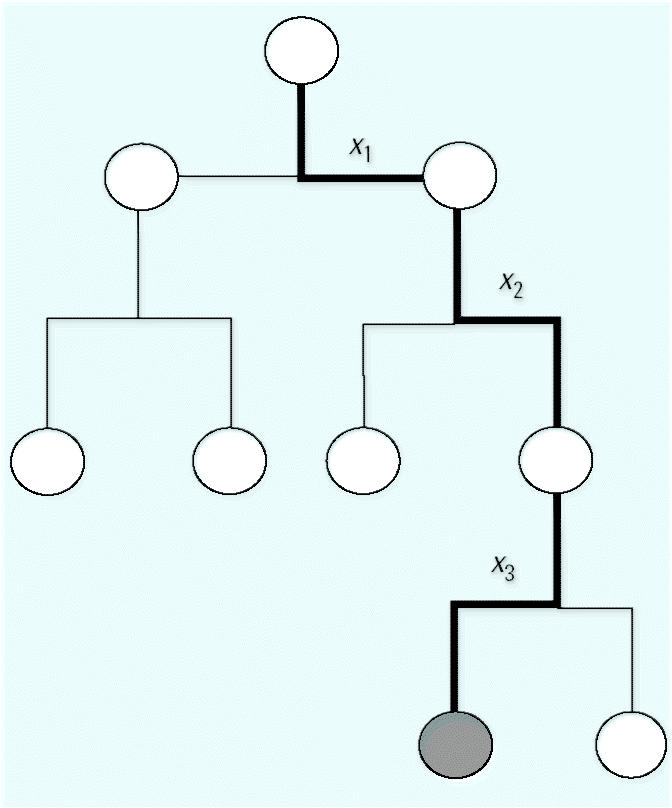
Schematic illustration for defining the training domain of a tree. For an unknown chemical predicted by the tree, its classification is determined by the terminal node (dark circle) to which it belongs. There are three descriptors used in the path (bold line) from the root to the terminal node and the range of these three descriptors across all chemicals in the training set determines the training domain.

Suppose there is a forest that contains *n* trees (*i* =1,…*n*). For the *i^th^* tree, the classification of an unknown chemical is determined by only one terminal node that is descendent from the root node through a set of “IF-THEN” rules based on *k* descriptors *x_ij_* (*j*=1,…*k*) (Figure 2). Let *x_ij_*(max) and *x_ij_*(min) denote the maximum and minimum values for *x_ij_* across the entire data set and *y_ij_* denote the descriptor values of the unknown chemicals corresponding to *x_ij_*. If *y_ij_* is either > *x_ij_*(max) or <*x_ij_*(min), then it is outside the range of the training domain defined by *x_ij_* in the “IF-THEN” rule in the path from the root to the terminal node in the *i^th^* tree. Thus, the distance beyond the training domain for the unknown chemical in the tree *i* can be calculated by *d_ij_* = |*y_ij_* − *x_ij_*(max)| if *y_ij_* > *x_ij_*(max), *d_ij_* = |*y_ij_* − *x_ij_*(min)| if *y_ij_* < *x_ij_*(min), or *d_ij_* = 0 if *y_ij_*> *x_ij_*(min) or *y_ij_* < *x_ij_*(max) (within the training domain). For the forest, the total percentage of extrapolation outside the training domain is:


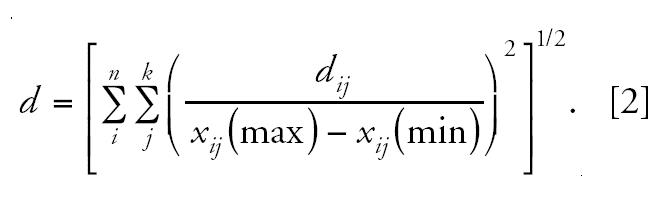


The prediction accuracy within domain *d* is calculated by dividing correct predictions by total number of chemicals in this extrapolated domain.

### Cross-Validation for Assessing Prediction Confidence and Domain Extrapolation

We used 10-fold cross-validation to assess a forest’s prediction accuracy for unknown chemicals in different domains of prediction confidence and extrapolation. In this procedure the data set is randomly divided into 10 equal portions, and each portion is excluded once and predicted by the forest produced using the remaining nine portions to train the model. Because the 10-fold cross-validation results vary for each run due to random partitioning of the data set, we repeated the process 2,000 times. The average result of the multiple cross-validation runs provides an unbiased assessment of a forest for predicting unknown chemicals with respect to prediction confidence and extrapolation sensitivity.

## Results

Table 1 summarizes the fitting results of the forests for both the ER232 and ER1092 data sets. The forests had concordances around 95% with high specificity and sensitivity.

Since a statistically sound fitted model provides limited indication of its capability for predicting chemicals that are not included in training, we applied 2,000 runs of 10-fold cross-validation to assess the prediction confidence and extrapolation sensitivity of the model for predicting unknown chemicals.

**Figure 3:**
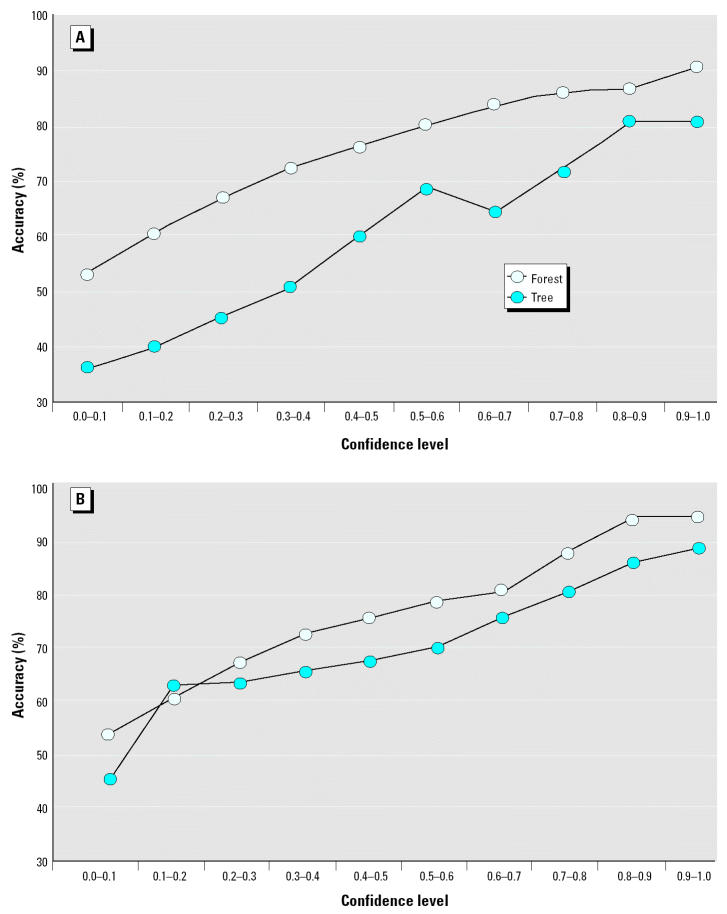
Prediction accuracy versus confidence level. Data were calculated from 2,000 runs of 10-fold cross-validation for (*A*) ER232 and (*B* ) ER1092.

Figure 3 plots forest prediction accuracy versus prediction confidence for ER232 (Figure 3A) and ER1092 (Figure 3B), respectively. For comparison, the results of the first tree in each forest are also plotted in Figure 3. It is readily apparent that the forests have substantially higher prediction accuracy than the tree across the entire range of confidence levels. Importantly, there is a strong trend of higher accuracy with increasing confidence level. We arbitrarily defined two confidence regions, HC and LC corresponding to confidence levels > 0.4 and < 0.4, respectively. Table 2 compares the HC, LC, and overall prediction accuracy. The HC prediction accuracy is approximately 86%, about 22% higher than the prediction accuracy for the LC regions (~ 64%). There is about 5–7% higher prediction accuracy for the HC regions than for the overall prediction accuracy (Table 2). The HC predictions account for approximately 80% of chemicals for ER232 and approximately 70% for ER1092.

**Figure 4:**
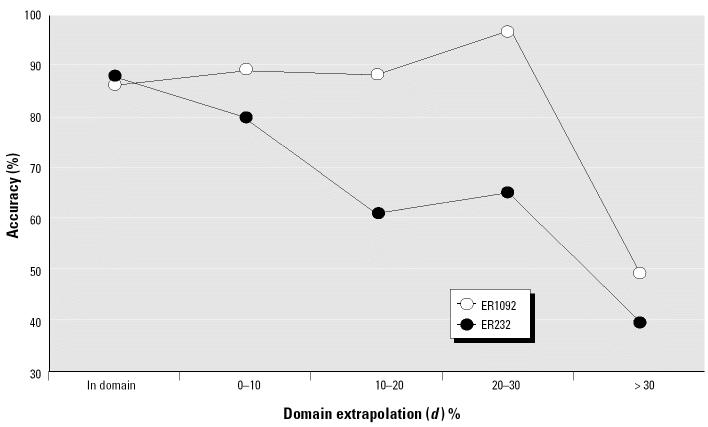
Prediction accuracy in different domains of extrapolation for ER232 and ER1092 from 2,000 runs of 10-fold cross-validation.

On the basis of the same cross-validation results, we also assessed the prediction accuracy for the chemicals as a function of extrapolation outside the training domain. Figure 4 compares for both ER232 and ER1092 the overall prediction accuracy for chemicals within the domain defined by the training set chemicals with accuracy for chemicals falling several degrees of extrapolation outside the training domain, as defined by Equation 2. Generally, the farther away the chemicals were from the training domain, the more loss in prediction accuracy was observed. For ER232 the prediction accuracy was reduced by some 10% for chemicals with a 10% extrapolation. In contrast for ER1092, a major decrease in accuracy only occurred beyond a 30% extrapolation.

Table 3 further breaks down the overall prediction accuracy shown in Figure 4 into the accuracies for the HC and LC regions and also gives the distribution of predictions within the extrapolated domains. For the HC prediction region the trend of decreasing prediction accuracy with increasing extrapolation is consistent with Figure 4 for both ER232 and ER1092. In the HC region for both data sets, prediction accuracy is comparable when extrapolation does not exceed 10%. Prediction accuracy declines more notably for chemicals with > 10% extrapolation for ER232 (some with > 16%), and for chemicals with > 30% extrapolation for ER1092. In contrast the LC region prediction accuracy is consistently lower, as expected, and exhibits no discernable trend with extent of extrapolation.

## Discussion

We used the novel QSAR method DF to develop two classification models to predict ER binding. Such models could be important in prioritizing chemicals for testing based on likelihood of activity. We furthermore objectively and quantitatively assessed the applicability domains of the models by computing prediction confidence and domain extrapolation for predicting unknown chemicals with an extensive cross-validation. We found that accuracy in classifying unknown chemicals is dependent on both prediction confidence and domain extrapolation, with the dependence most pronounced for prediction confidence. The prediction accuracy is notably higher for the chemicals in the HC domain than for those in the LC domain. In the HC domain, the forest model based on the large data set ER1092 is much better able to extrapolate outside the structural domain defined by the training data than is the forest model based on the small data set ER232 and specifically by some 30% compared with 10%. We propose that the ER1092 model is most suitable for aiding in prioritizing chemicals for testing as possible EDCs.

The consistently lower prediction accuracy in the LC domain compared to that of the HC domain seems minimally affected by the extent of extrapolation. For many repeated runs of cross-validation with random partitioning, chemicals in the HC domain average 70–80% of the total for both data sets. It should be noted that the distribution of the chemicals between the high and low confidence regions could vary when applying the model to a test set. Actual distribution depends largely on how well the training set represents the test set chemicals. In the cross-validation, however, the proportion of chemicals in the HC domain is sensitive to the structural diversity and quality of the training set.

The ability to quantify confidence greatly enhances the utility of any classification or QSAR method. The ability to accurately gauge confidence of predictions may also determine how best to apply the model. For example, considering the forest models presented here for use in screening and testing for potential EDCs, the HC and LC domain predictions could be used in separate ways. Chemicals in the HC domain are candidates for applying more rigorous quantitative models (Shi et al. 2001) to calculate binding affinities that are, in turn, used to rank-order chemicals for experimental evaluation. However, for chemicals in the LC domain, more thorough evaluation based on other types of models (Hong et al. 2002; Shi et al. 2002; Tong et al. 2003a) and/or assays should be required.

Validation is an important step in developing a useful QSAR model. There are two common validation methods— cross-validation and external validation (Tong et al. 2003c). For most classification methods, descriptor selection is normally executed prior to model training. Without preselection of the descriptor variables, the computational expense of cross-validation could be prohibitive. However, preselection of descriptors also constitutes a bias, suggesting that cross-validation may overestimate a model’s true predictive accuracy for unknown chemicals. For such cases preselecting an external test set not used in training becomes critical to estimating predictive accuracy. But, setting aside an external test set detrimentally reduces the size of the available training set, resulting in the loss of data that would likely improve the model. Ideally, the external test set would be rationally selected to represent the chemicals to which the model would be applied. In reality, however, because of the difficulty of such a task, we are unaware of any model development and test set selection in the literature that incorporates a systematic selection of a representative test set.

Bias in descriptor selection is not a factor in DF, where in each step of the cross-validation a new set of descriptors is selected that forms the best forest to represent each random spilt between training and testing data. The full integration of variable selection with forest construction means that the cross-validation accuracy is more likely to represent the true predictivity. Of course, a prediction test on external data is always desirable because it is a real-world application of the model, but very rarely is sufficient data available to warrant complete exclusion of some data from the training data. In a sense, cross-validation closely resembles the conduct of multiple tests on external data. Thus, we choose a rigorous and extensive cross-validation method to validate the models’ predictivities in this study, which is able to assess many possible partitions of the training and test sets and then can provide an unbiased and objective means for assessing a model’s quality.

A large number of QSAR models for ER binding are reported in the literature (Bradbury et al. 1996; Sadler et al. 1998; Waller et al. 1996; Wiese et al. 1997; Zheng and Tropsha 2000), including our own (Tong et al. 1997a, 1997b, 1998; Xing et al. 1999). Although these models yield good statistical results, none explicitly address and assess the confidence in predicting unknown chemicals. We demonstrated in this study that there could be more than a 22% difference in prediction accuracy for the chemicals with high confidence compared with those with low confidence. Thus, for practical applications, having prediction confidence together with the actual predictions greatly extends the usefulness of QSAR and classification models. In regulatory application, the justification for using such models may very well depend on having measures of confidence in the predictions.

Four types of uncertainty are generally recognized as affecting the prediction confidence of a QSAR model (Tong et al. 2003c), and all generally are dependent on either the nature of the data set or the choice of the statistical algorithm. First, predictions from any model are intrinsically no better than the experimental data employed to develop the model. Any limitations of the assay used to generate the training data equally extends to the model’s predictions. Second, commonly employed statistical methods vary in their abilities to appropriately capture the functional relationship of structural descriptors and activity. Third, for classification models specifically, class assignment is sensitive to a defined cutoff value to distinguish active from inactive. As the cutoff value is lowered, it is likely that the error will increase, even for a well-designed and well-executed assay. The increased experimental error in close proximity to the cutoff value will be transferred to the classification model, which in turn will increase false prediction rate for chemicals with activity in this region. Fourth, a chemical can be represented by different types of descriptors. We often find that, even for a simple mechanism such as ER-binding, some descriptors may well represent binding dependencies for one structural class, whereas other features will better represent binding dependencies for a different structural class. In such cases, regardless of how rigorously the validation procedure is employed, the model may give incorrect predictions for some chemicals, as the entire chemistry space of active chemicals is unknown. These four types of uncertainty determine the applicability domain of a QSAR model, and adequate assessment of this domain that bounds and guides the model’s usage, especially in regulatory application, is paramount. The assessment procedure proposed in this study should be equally applicable to other QSAR methods.

## Figures and Tables

**Table 1:** Statistics of the forest models based on ER232 and ER1092.

	ER232	ER1092
Number of chemicals	232	1092
Number (%) of misclassifications	5 (2.16%)	50 (4.58%)
Number of trees combined	6	4
Number of descriptors used	79	138
Accuracy	96.6%	95.4%
Specificity	96.0%	91.0%
Sensitivity	96.9%	97.6%

**Table 2:** The HC and LC predictions from 2,000 runs of 10-fold cross-validation for ER232 and ER1092.

	ER232		ER1092	
Confidence regions	Accuracy (%)	Percentage of chemicals	Accuracy (%)	Percentage of chemicals
HC	86.6	79.2	86.3	69.9
LC	63.8	20.8	64.7	30.1
All	81.9	100	79.7	100

Abbreviations: HC , high confidence; LC, low confidence.

**Table 3:** Prediction accuracy in different regions of confidence and extrapolation derived from 2,000 runs of 10-fold cross-validation for ER232 and ER1092.

		HC region		LC region	
Data set	Extrapolation [(*d*) %]	Accuracy (%)	No. of predictions	Accuracy (%)	No. of predictions
ER232	0	87.8	349,595	64.7	83,393
	0–10	79.7	10,442	55.8	6,216
	10–20	61.1	1,325	50.5	2,651
	20–30	65.3	853	63.9	1,086
	> 30	39.7	5,614	65.9	2,825
ER1092	0	86.4	1,511,180	64.4	645,177
	0–10	89.4	6,896	61.5	5,135
	10–20	88.5	3,914	68.1	3,453
	20–30	96.8	1,209	75.3	959
	> 30	48.9	3,560	54.1	2,517
